# Association between Resistant Arterial Hypertension, Type 2 Diabetes, and Selected microRNAs

**DOI:** 10.3390/jcm13020542

**Published:** 2024-01-18

**Authors:** Robert Błaszczyk, Alicja Petniak, Jacek Bogucki, Janusz Kocki, Andrzej Wysokiński, Andrzej Głowniak

**Affiliations:** 1 Department of Cardiology, Medical University of Lublin, 20-090 Lublin, Poland; andrzej.wysokinski@umlub.pl (A.W.); andrzej.glowniak@umlub.pl (A.G.); 2 Department of Clinical Genetics, Medical University of Lublin, 20-080 Lublin, Poland; alicja.petniak@umlub.pl (A.P.); janusz.kocki@umlub.pl (J.K.); 3 Department of Organic Chemistry, Medical University of Lublin, 20-093 Lublin, Poland; jacek.bogucki@umlub.pl

**Keywords:** resistant arterial hypertension, diabetes type 2, microRNA

## Abstract

Introduction: In recent years, a very close relationship between miRNA and cardiovascular diseases has been found. RAH and T2DM are accompanied by a change in the microRNA expression spectrum. Objectives: This study aimed to evaluate the clinical characteristics and expression of selected microRNAs in patients with idiopathic RAH and T2DM. Patients and methods: A total of 115 patients with RAH were included in this study. Among them were 53 patients (46.09%) with T2DM. miRNA levels were determined using quantitative real-time polymerase chain reaction. The expression of the examined genes was calculated from the formula RQ = 2^−ΔΔCT^. Results: Analysis using the Mann–Whitney U test showed a statistically significant (*p* < 0.05) difference in the expression of MIR1-1 (*p* = 0.031) and MIR195 (*p* = 0.042) associated with the occurrence of T2DM in the subjects. The value of MIR1-1 gene expression was statistically significantly higher in patients with T2DM (median: 0.352; mean: 0.386; standard deviation: 0.923) compared to patients without T2DM (median: 0.147; mean: −0.02; standard deviation: 0.824). The value of MIR195 gene expression was statistically significantly higher in patients with T2DM (median: 0.389, mean: 0.442; standard deviation: 0.819) compared to patients without T2DM (median: −0.027; mean: 0.08; standard deviation: 0.942). Conclusions: The values of MIR1-1 and MIR195 gene expression were statistically significantly higher in patients with RAH and T2DM compared to patients with RAH and without T2DM. Further studies are necessary to precisely clarify the roles of miRNAs in patients with RAH and T2DM. They should demonstrate the utility of these genetic markers in clinical practice.

## 1. Introduction

Arterial hypertension (AH) is a disease that is caused by a combination of genetic susceptibility and environmental factors. It is a well-established fact that AH is a major contributor to cardiovascular and cerebrovascular diseases, with a global prevalence projected to increase from 26.4% in 2000 to 29.2% in 2025 [1]. The pathogenesis of AH is caused by several mechanisms, which are of utmost importance. These mechanisms include increased activity of the sympathetic nervous system, overactivation of the renin–angiotensin–aldosterone system (RAAS), dysfunction of the vascular endothelium, impaired platelet function, thrombogenesis, vascular smooth muscle, cardiac hypertrophy, and altered angiogenesis [2]. According to the latest definition, resistant arterial hypertension (RAH) is diagnosed when systolic blood pressure (SBP) and diastolic blood pressure (DBP) values are ≥140 mm Hg and ≥90 mm Hg, respectively, despite the use of three antihypertensive drugs in the maximum recommended and tolerated doses: an inhibitor of the RAAS system (angiotensin-converting enzyme inhibitor—ACE inhibitor or receptor antagonist AT1—sartan), long-acting calcium channel blocker, and thiazide/thiazide-like diuretics. In addition, two more conditions must be met for the diagnosis of RAH: 1. abnormal blood pressure control confirmed by ambulatory blood pressure monitoring (ABPM)/home blood pressure monitoring (HBPM) (if the ABPM method is not available to confirm RAH, HBPM is recommended) and 2. causes of pseudo resistance have been excluded—especially patient non-compliance with medical recommendations and secondary forms of hypertension [3]. Refractory hypertension occurs when blood pressure remains uncontrolled despite the use of five or more antihypertensive drugs from different classes, even when used at maximum or near-maximum dosages. Patients with RAH have a higher risk of developing chronic kidney disease and premature cardiovascular events [4,5]. The incidence of RAH and refractory hypertension is difficult to estimate. The ESH 2023 guidelines indicate that, despite significant differences in individual clinical trials, the actual incidence of true RAH in the general population of treated patients with hypertension is approximately 5%. However, pseudo resistance affects up to 10–20% of patients with hypertension [3,6].

The complex nature of type 2 diabetes (T2DM) and metabolic disorders such as hyperglycemia, hyperinsulinemia, insulin resistance, excess fatty acids, and pancreatic beta cell dysfunction can lead to thrombosis, vasoconstriction, vascular inflammation, and atherogenesis. This ultimately results in the development of RAH. Additionally, over 39% of newly diagnosed T2DM patients have higher blood pressure than those without diabetes, and up to 75% of adult T2DM patients develop AH [7,8]. Considering the early occurrence of functional and structural changes in the cardiovascular and cerebrovascular systems, T2DM is associated with a higher rate of RAH incidence. Even though there have been advancements in diagnosing and treating patients with AH and T2DM, and despite the continuous updating of guidelines with new scientific data, there is still a need for more precise clinical therapeutics. This is mainly due to the lack of new, non-invasive biomarkers and the identification of therapeutic targets.

Patients who suffer from both AH (RAH) and T2DM may experience several related disorders, which can mutually intensify and elevate the risk of cardiovascular complications. These disorders include a higher degree of endothelial dysfunction, reduced plasma nitric oxide (NO) levels due to a suppressed L-arginine/endothelial nitric oxide synthase (eNOS) pathway, an increased level of reactive oxygen species (ROS), increased leukocyte adhesion to the vascular wall, and an inflammatory response in the vasculature.

It is well-known that 98.5% of the human genome consists of non-protein-coding DNA. However, some of this non-protein-coding DNA can be transcribed to a group of functional RNA species known as non-coding RNA (ncRNA). ncRNA is classified into three categories based on its size: small (19–25 nucleotides), intermediate-sized (20–200 nucleotides), and long (above 200 nucleotides). The best-known microRNAs are small, covalently closed, single-stranded RNAs that direct the posttranscriptional repression of mRNA targets. The biogenesis of miRNAs involves several phases, such as the transcription of primary miRNAs (pri-miRNAs), nuclear Drosha-mediated processing, cytoplasmic Dicer-mediated processing, and loading onto Argonaute (Ago) proteins [9,10,11,12]. In recent years, it has been discovered that miRNA has a very close relationship with cardiovascular diseases. Both AH and T2DM are accompanied by a change in the microRNA expression spectrum. miRNAs play an important role in various biological processes, such as necrosis, apoptosis, cell development and differentiation, and signaling. The dysregulation of these processes can cause various diseases like AH and T2DM.

Because the pathogenesis of AH and T2DM is extremely complex and not fully understood, both diseases share common pathophysiological elements and complications. The above-mentioned pathophysiological pathways are regulated by microRNAs, so it seems reasonable to assess the expression of microRNAs in these diseases.

## 2. Patients and Methods

This study aimed to evaluate the clinical characteristics and expression of selected microRNAs in patients with idiopathic RAH. This study was conducted in 2021 at the Department of Cardiology of the Medical University of Lublin. First, a clinical examination was performed; next, blood samples were taken for genetic tests. This study was conducted following the principles and guidelines of the Declaration of Helsinki and approved by the Bioethics Committee at the Medical University of Lublin (Decision No. KE-0254/141/2020). All blood samples were collected from participants after obtaining written informed consent.

### 2.1. Cell Isolation

Isolation of PBMCs was conducted using Ficoll-Paque Plus™ via density gradient centrifugation from whole blood samples (Cytiva, Uppsala, Sweden). Whole-blood samples obtained from study individuals were collected into 2.7 mL S-Monovette vials (Sarstedt, Germany) containing K3 ethylenediaminetetraacetic acid (EDTA) as an anticoagulant factor. Samples were directly submitted to PBMC isolation via density gradient centrifugation using Ficoll-Paque Plus™ (Cytiva, Uppsala, Sweden). Whole-blood samples were diluted in equal volumes of PBS (phosphate-buffered saline, without calcium and magnesium ions, Biomed-Lublin, Lublin, Poland) in sterile 15 mL Eppendorf conical tubes (Eppendorf, Germany). Diluted blood was carefully layered on 3 mL Ficoll-Paque Plus™ in a new, sterile, 15 mL conical tube. Tubes were centrifuged at 400× *g* for 30 min at 20 °C (5810 R Centrifuge, Eppendorf, Germany), and the brake was turned off. The layer of mononuclear cells was transferred to a new, sterile, 15 mL Eppendorf conical tubes. Cells were gently resuspended in 3 volumes of PBS by pipetting. The tubes were centrifuged at 450× *g* for 10 min at 20 °C (5810 R Centrifuge, Eppendorf, Germany). The supernatant was removed. This step was repeated twice. The cell pellet was resuspended in 1 mL of PBS. The entire volume was distributed to several sterile 1.5 mL centrifuge tubes (DNA LoBind Tube, Eppendorf, Germany) and centrifuged at 400× *g* for 10 min at 4 °C (5415 R Centrifuge, Eppendorf, Germany). After the last centrifugation, the supernatant was removed and the tubes with PBMC pellets were stored at −80 °C till the isolation of RNA.

### 2.2. Total RNA Isolation

Total RNA was isolated from PBMCs using the mirVana™ miRNA Isolation Kit (Invitrogen by Thermo Fisher Scientific, Vilnius, Lithuania), according to the manufacturer’s protocol. The quantity and quality assessment of isolated total RNA was performed using a NanoDrop 2000c Spectrophotometer (Thermo Fisher Scientific, Waltham, MA, USA). For all samples analyzed, the A260/A280 ratio was between 1.8 and 2.0.

### 2.3. Reverse Transcription Reaction

The reverse transcription process was performed according to the manufacturer’s instructions using the TaqMan MicroRNA Reverse Transcription Kit and miRNA-specific stem-loop primers (Applied Biosystem, Vilnius, Lithuania). The reverse transcription reaction was carried out in a volume of 15 μL, consisting of 1.5 μL 10× reverse transcription buffer, 0.15 μL 100 mM dNTPs (with dTTP), 3 μL 5× RT primer, 0.19 μL RNase inhibitor, 20 U/µL, 1 μL MultiScribe™ Reverse Transcriptase, 50 U/µL, 4.16 μL nuclease-free water, and 5 μL of total RNA (10 ng total RNA dissolved in 5 μL nuclease-free water). cDNA synthesis was performed on a Veriti Dx Thermal Cycler (Applied Biosystems, Foster City, CA, USA) in the following order: phase I, 16 °C—30 min; phase II, 42 °C—30 min; stop reaction, 85 °C—5 min. The resulting cDNA was used for real-time PCR.

### 2.4. qPCR Reaction

qPCR reactions were carried out with the use of 0.67 μL of RT product along with 3.83 μL of nuclease-free water, 0.5 μL of miRNA-specific primer/probe mix, and 5 μL TaqMan™ Universal Master Mix II with UNG (Applied Biosystems, Vilnius, Lithuania). Quantitative real-time PCR was performed in triplicate in a StepOnePlus real-time PCR system (Applied Biosystems, Waltham, MA, USA) using standard TaqMan^®^ microRNA assays (Applied Biosystems, Pleasanton, CA, USA): MIR1-1 (hsa-miR-1: assay ID 002222), MIR21 (hsa-miR-21: assay ID 000397), MIR26B (hsa-miR-26b: assay ID 000407), MIR126 (hsa-miR-126b: assay ID 000451), MIR133A1 (hsa-miR-133a: assay ID 002246), MIR143 (hsa-miR-143: assay ID 002249), MIR145 (hsa-miR-145: assay ID 002149), MIR155 (hsa-miR-155: assay ID 002623), MIR195 (hsa-miR-195: assay ID 000494), MIR208A (hsa-miR-208: assay ID 000511), MIR320A (hsa-miR-320: assay ID 002277), and SNORD48 (RNU48: assay ID: 001006) as a control. The reactions were incubated in a 96-well MicroAmp Fast Optical 0.1 mL reaction plate at 50 °C for 2 min and 95 °C for 10 min, followed by 40 cycles of 95 °C for 15 s and 60 °C for 60 s.

The expression of the examined genes was calculated from the formula RQ = 2^−ΔΔCT^ [13]. RNU48 snRNA was used as a normalizer during the analysis. Expression Suite Software 1.0.3 was used to calculate the gene expression level (Life Technologies, Waltham, MA, USA).

### 2.5. Statistical Analysis of Data

Statistica v.13.3 was used in the statistical analysis and graphic design (*p* < 0.05 was assumed statistically significant). The histograms and Shapirro-Wilk test were used to check the normality of the data distributions. Because the distributions of the analyzed variables deviated from the normal distributions, non-parametric statistical methods were used. The U Mann–Whitney test was used to calculate the differences in the expression level and the r-Spearman coefficient was used for correlation analysis.

## 3. Results

Baseline characteristics regarding demographics, biochemical parameters, comorbidities, and echocardiography parameters were collected. The Chronic Kidney Disease Epidemiology Collaboration (CKD-EPI) equation was used to calculate the estimated glomerular filtration rate (eGFR) and assess patients’ kidney function. Each participant underwent a routine echocardiographic examination according to the standard protocol.

In total, 115 patients with RAH were included in the study. Among them were 53 patients (46.09%) with type 2 diabetes. The mean age was 65.67 (±11.15) years old. There were more women (53.91%) than men in the study group. The average duration of AH in the study group was 10.77 (±4.80) years, while the average duration of RAH was 4.12 (±1.68) years. The most common comorbidities included coronary artery disease—80 patients (69.57%), PCI history—39 patients (33.91%), diagnosed CKD—40 patients (34.78%), paroxysmal AF—21 patients (18.26%), and history of TIA—21 (18.26%). Among echocardiographic parameters, the mean LVEF was 59.72% (±5.81), LA volume—94.73 mL (±19.25), LAVI—46.28 mL (±10.89), LVMI—110.68 mL (±21.68), LVMM—216.22 mL (±46.78), and RWT—0.46 mL (±0.05). Table 1 shows the baseline characteristics of the studied group.

The patients included in this study were treated with pharmacotherapy according to the recommendations of the European Society of Hypertension and the European Society of Cardiology [3,14]. All groups of hypertensive drugs were administered. Table 2 shows the pharmacotherapy of RAH.

This study included 28 women (52.83%) and 25 men (47.17%) with RAH and T2DM. Table 3 and Table 4 show the characteristics of the study group divided into two subgroups: RAH without T2DM and RAH with T2DM.

When comparing patients with RAH and T2DM and without T2DM, the more frequent occurrence of atrial fibrillation in the RAH group without T2DM is noteworthy. More than 90% of patients with RAH and T2DM had diagnosed chronic heart failure with preserved left ventricular ejection fraction (HfpEF), while in the RAH group without T2DM, this percentage was 74.19%. In the case of cerebrovascular disease, a higher percentage of patients with previous stroke history was noted in the RAH and T2DM group (16.98% vs. 12.90%). Also in the RAH and T2DM group, there was a higher percentage of patients with a history of myocardial infarction (22.64% vs. 8.06%), PCI (45.28% vs. 24.19%), and CABG (15.09% vs. 1.61%). Table 5 shows cardiovascular morbidity in the studied patient groups.

The analysis showed that patients with RAH and T2DM had higher values of the parameters of left atrial enlargement (LA dimension in LAX: 40 mm vs. 39.42 mm; LA surface area in Ap4CH: 21.72 mm vs. 20.75 mm; LA volume: 99.41 mL vs. 90.74 mL, LAVI: 48.08 mL/m^2^ vs. 44.74 mL/m^2^) and left ventricle hypertrophy (LVMI: 113 g/m^2^ vs. 108.69 g/m^2^, LVMM: 221.37 g vs. 211.81 g, RWT: 0.4560 vs. 0.4565) compared to patients with RAH and without T2DM (Table 6 and Table 7). In the group of patients with RAH and T2DM, a higher percentage of diastolic dysfunction was found (92.45%) compared to patients with RAH without T2DM (77.41%).

### Statistical Analysis

**Figure 1:**
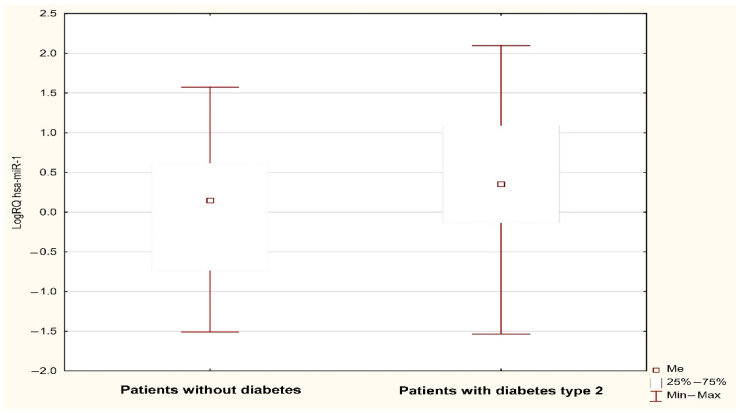
LogRQ MIR-1-1 gene expression value in non-diabetic and type 2 diabetes patients.

**Figure 2:**
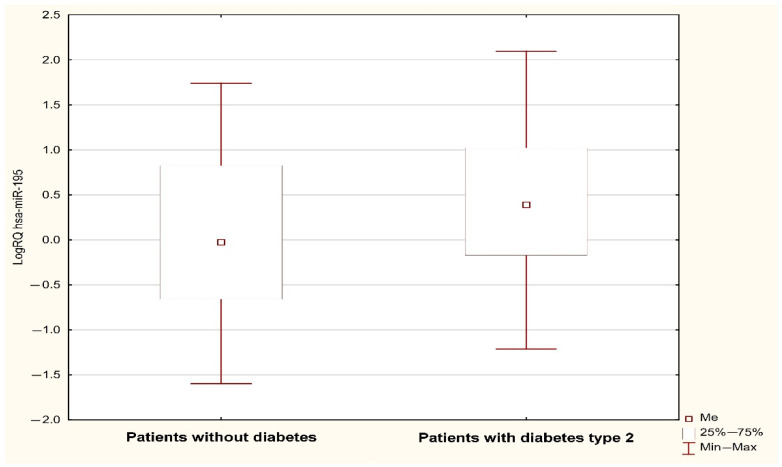
LogRQ MIR195 expression value in non-diabetic and type 2 diabetes patients.

Analysis using the Mann–Whitney U test showed a statistically significant (*p* < 0.05) difference in the expression of MIR1-1 (*p* = 0.031) and MIR195 (*p* = 0.042) associated with the occurrence of type 2 diabetes in the subjects. The value of MIR1-1 gene expression was statistically significantly higher in patients with type 2 diabetes (median: 0.352; mean: 0.386; standard deviation: 0.923) compared to patients without type 2 diabetes (median: 0.147; mean: −0.02; standard deviation: 0.824) (Figure 1). The value of MIR195 gene expression was statistically significantly higher in patients with type 2 diabetes (median: 0.389, mean: 0.442; standard deviation: 0.819) compared to patients without type 2 diabetes (median: −0.027; mean: 0.08; standard deviation: 0.942) (Figure 2). A possible trend toward significance was found in the expression of the MIR126 gene (*p* = 0.051), the MIR133A1 gene (*p* = 0.076), the MIR143 gene (*p* = 0.08), and the MIR320A gene (*p* = 0.095) in patients with type 2 diabetes. The value of MIR126 gene expression showed a statistical trend toward significance in patients with type 2 diabetes (median: 0.332; mean: 0.427; standard deviation: 0.753) compared to patients without type 2 diabetes (median: 0.112; mean: 0.116; standard deviation: 0.798). The value of MIR133A1 gene expression showed a statistical trend toward significance in patients with type 2 diabetes (median: 0.147; mean: 0.233; standard deviation: 1.056) in contrast to patients without type 2 diabetes (median: −0.168; mean: −0.106, standard deviation: 0.883). The value of MIR143 gene expression showed a statistical trend toward significance in patients with type 2 diabetes (median: 0.547; mean: 0.494; standard deviation: 1.047) compared to patients without type 2 diabetes (median: 0.205; mean: 0.071; standard deviation: 1.148). The value of MIR320A gene expression showed a trend close to significance in patients with type 2 diabetes (median: 0.045; mean: 0.144; standard deviation: 0.786) in contrast to patients with no diagnosis of type 2 diabetes (median: −0.210, mean: −0.139; standard deviation: 0.844). In the case of the remaining microRNAs (MIR145, MIR155, MIR21, MIR26b, and MIR-208A), the differences between the two study groups were statistically insignificant.

The statistical analysis carried out showed no difference in the value of miR (hsa-miR-1, hsa-miR-126*, hsa-miR-133a, hsa-miR-143, hsa-miR-145*, hsa-miR-155, hsa-miR-195, hsa-miR-208, hsa-miR-21, hsa-miR-26b, hsa-miR-320) expressions in women and men (Table 8).

Additionally, a Spearman test was performed. In patients with RAH who had not been diagnosed with type 2 diabetes, there was a negative correlation between the duration of RAH and the expression of miR-145 and miR-208 (r Spearman = −0.306, *p* < 0.05; r Spearman = −0.290, *p* < 0.05).

## 4. Discussion

miRNAs play a crucial role in many physiological and pathological processes and regulate various functions in the cardiovascular system, kidneys, and liver. However, the specific targets of most microRNAs are still not well understood. These microRNAs can repress and inhibit the expression of genes. Even subtle changes in the levels of miRNAs can disrupt biochemical and physiological pathways, leading to the onset of various disorders.

In the literature, data confirm the relationship between MIR1, MIR195, and the coexistence of AH and T2DM. MIR195 plays an important role in regulating cardiovascular diseases. In a recently published work, Hao et al. showed the role of MIR195a-5p in blood pressure regulation via electrolyte excretion involving Na^+^-K^+^-2Cl^−^ cotransporter isoform A (NKCC2A). They suggested that MIR195a-5p is involved in the mechanism by which tumor necrosis factor-α (TNF-α) restricts increases in sodium retention and blood pressure that are dependent on NKCC2A after a rise in salt consumption. NKCC2A expression follows in the thick ascending limb of the Henle loop (TAL). TNF increases MIR195a-5p expression, which in turn interacts with the 3′UTR of NKCCA2 to regulate NaCl excretion. This attenuates NaCl-induced increases in blood pressure [15]. One of the most common complications of improperly controlled AH (RAH) is cardiac hypertrophy. MIR195 plays a significant role in this case. Zheng et al. showed that inhibited MIR195-5p expression attenuated hypertrophy in streptozocin-induced diabetic mice. In their study, overexpression of MIR195-5p significantly promoted AngII-induced cardiac hypertrophy. Therapeutic silencing of MIR195 improves myocardial function in diabetes by reducing oxidative damage, inhibiting apoptosis, and promoting endothelial cell angiogenesis. The authors concluded that MIR195 may represent an alternative therapeutic target for diabetic heart diseases [16]. Previous publications have shown that MIR195 inhibits the gene expression of B cell leukemia/lymphoma 2 (BCL-2) and Sirtuin 1 (SIRT1) in cardiomyocytes, leading to decreased levels of these proteins in diabetic hearts. Decreased MIR195 expression increased levels of BCL-2 protein, which is a known anti-apoptotic protein. SIRT1, which is activated by resveratrol, causes improved cardiac function in animal models of diabetes. MIR195 can contribute to diabetic cardiomyopathy by affecting systemic inflammation, insulin sensitivity, and causing liver damage [17,18]. BCL-2 also regulates another important anti-apoptotic protein—heat shock protein 60 (Hsp60). Increased BCL-2 expression levels lead to decreased Hsp60 levels in the myocardium of diabetic rats. MIR1 plays an important role in inducing high glucose-based apoptosis of cardiac cells [19]. In an interesting study, it was found that MIR1 mediates insulin-like growth factor (IGF-1), which is a well-known anti-apoptotic factor. When H9C2 cells were administered, high glucose, mitochondrial dysfunction, and apoptosis were observed due to high expression of MIR1. However, IGF-1 blocked these effects by regulating MIR1 expression [20]. In another publication, Liu et al. investigated the effect of MIR1 expression on vascular smooth muscle cells (VSMCs). Prolonged high blood pressure can cause mechanical stretch of the vascular wall, leading to changes in VSMCs. These changes may include increased proliferation, synthesis of collagen and extracellular matrix, migration of cells, and decreased expression of contractility markers, which are typically present in this type of muscle cell. MIR1 inhibits the proliferation of muscle cells such as cardiomyocytes. In the above-mentioned publication, the downregulation of miR1 was responsible for a higher rate of VSMC proliferation. The anti-proliferative effects of MIR1 are mediated through IGF-1 [21,22].

Hu Y. et al. studied the relationship between MIR195 expression and Dopamine receptor D1 function (DRD1) in a group of patients with AH and T2DM. This receptor belongs to the superfamily of G-protein-coupled receptors (GPCRs). The primary importance of this subtype receptor is sodium reuptake and renal sodium excretion. Dysfunction of DRD1 leads to essential hypertension. MIR195-5p was found to efficiently suppress DRD1 expression by binding to the two 3′UTRs. The study also identified two single nucleotide polymorphisms (SNPs), namely 231T-A and 233C-G, in the MIR195-5p binding sites of the DRD1 3′-UTR. The results suggest the potential clinical significance of DRD1 regulation by MIR195-5p in patients with AH and concurrent type 2 diabetes (T2DM) [23].

Data from the literature point to the fact that up-regulation of MIR195 contributes to cardiac hypertrophy-induced arrhythmia by targeting calcium and potassium channels. miR-195 can directly target the CACNB1 gene (calcium voltage-gated channel auxiliary subunit beta 1), KCNJ2 gene (potassium voltage-gated channel subfamily J member 2), and KCND3 gene (potassium voltage-gated channel subfamily D member 3) to regulate Cavβ1, Kir2.1, and Kv4.3 protein domain expression. There are elevated levels of a certain substance, denoted by MIR195, in a failing heart muscle. These elevated levels regulate the acetylation of two important enzymes, pyruvate dehydrogenase (PDH) and ATP synthase. This in turn suppresses the activity of another important enzyme called Sirtuin 3 (SIRT3), which inhibits enzymatic activity. This presents a new pathway for research on MIR195. Xuan et al. have demonstrated that overexpression of MIR195 inhibits the expression of three proteins, namely Cavβ1, Kir2.1, and Kv4.3. However, when a MIR195 inhibitor is added, this downregulated protein expression is reversed [24,25].

In the study of arterial hypertension, cardiomyocyte apoptosis is closely related to AH. The balance between cell death and cell survival is a precisely controlled process. Accumulated data support the role of cardiomyocyte apoptosis in the development of various heart diseases. This includes the transition from hypertensive compensatory hypertrophy to heart failure [26]. Huang X et al. studied the relationship between MIRNA1 level and the expression of Bcl-2 and explored the role of MIRNA1 in cardiomyocytes in hypertensive rats. In their study, silencing MIR1 activated the mitogen-activated protein kinase (MAPK) pathway, promoted autophagy, and reduced the degree of myocardial fibrosis caused by hypertension [27]. 

In our study, in patients with RAH and without diagnosed T2DM, a negative correlation was found between the duration of RAH and the expression of miR-145 and miR-208. The role of MIR145 in the pathogenesis and regulation of AH (RAH) is extremely complex. Wang Y. et al. demonstrated the role of MIR145 in spontaneous hypertensive rats (SHRs) and rat vascular endothelial cells. Higher expression of MIR145 in hypertensive rats was associated with an important decrease in the production of nitric oxide. The direct target for MIR145 is solute carrier family 7 member 1 (SLC7A1). A decrease in the expression of MIR145 causes increased expression of SLC7A1 and phosphorylated endothelial nitric oxide synthase [28]. Chen et al. showed the role of complement 3 (C3) in the development of AH in SHRs. Simultaneous suppression of MIR145 and an increased expression of C3 induce the expression of Kruppel-like factor 5 (KLF5), which changes the type of VSMCs from the contractile to the synthetic phenotype. Furthermore, activation of C3 leads to the stimulation of liver X receptor α (LXRα), which in turn activates the renin–angiotensin system and platelet-derived growth factor A (PDGF-A) as well as transforming growth factor β1 (TGF-β1) in vascular smooth muscle cells (VSMCs) [29]. It also turns out that MIR145 may be responsible for VSMC remodeling through disintegrating and metalloprotease 17 (ADAM17), which is a protein involved in the shedding of several membrane proteins that are important for inflammation and immunity, namely TNF-α, intercellular adhesion molecule-1 (ICAM-1), and angiotensin convertase enzyme 2 (ACE2). A direct effect of ADAM17 on ACE2 has been demonstrated, leading to the shedding of ACE2 into the extracellular matrix. MIR145 attenuates the transformation of VSMCs from the contractile to synthetic type. Its molecular pathway includes the secretion of ACE2 in VSMCs mediated by ADAM17. Finally, the activation of the ACE2-Ang-(1-7)-Mas axis may yield vascular structural remodeling in metabolic hypertension [30].

There are also reports linking MIR208 with cardiac hypertrophy in patients with arterial hypertension. Huang X et al. examined the expression levels of MIR208 and sex-determining region Y-box 6 (SOX6) in patients with cardiac hypertrophy. The expression of MIR208 was significantly higher in patients with left ventricular hypertrophy (LVH) than in those without LVH. In an in vitro model, SOX6 expression levels were statistically significantly decreased in phenylephrine-stimulated cardiomyocytes compared to normal cardiomyocytes. The authors concluded that the expression levels of MIR208 are associated with LVH via negative regulation of SOX6 [31]. In our study, the parameters of left ventricle hypertrophy were more pronounced in the RAH and T2DM group.

Referring the study results to the studied population, it should be stated that patients with RAH and T2DM and increased expression of MIR1-1 and MIR-195 have increased cardiovascular morbidity, including the occurrence of heart failure and a history of stroke. An increased incidence of ischemic heart disease, including a number of myocardial infarctions and a history of PCI and CABG, was also observed in this group. The statistical analyses carried out so far showed no difference in the value of miR expressions in women and men.

## 5. Study Limitations

There are several limitations in our study. Firstly, in T2DM patients, muscle or adipose tissue samples would have been more relevant; however, we had no IRB approval for tissue sample collection. Secondly, it was a single-center study, performed on a relatively small number of patients; therefore, caution is needed when interpreting our findings with regard to the general RAH or T2DM populations. Thirdly, we used a statistical significance value of *p* < 0.05. Due to the possibility of false-positive results, a threshold of <0.0001 might have been preferable.

## 6. Conclusions

In conclusion, we found that the values of MIR1-1 and MIR195 gene expression were statistically significantly higher in patients with RAH and T2DM compared to patients with RAH and without T2DM. Further studies are necessary to precisely clarify the roles of miRNAs in patients with RAH and T2DM. They should also demonstrate the utility of these genetic markers in clinical practice.

## Figures and Tables

**Table 1:** Baseline characteristics of the studied group.

Parameter	Value (*n* = 112) M ± SD
Age	65.67 ± 11.15
Women	62 (53.91%)
Men	53 (46.09%)
BMI (kg/m^2^)	30.41 ± 4.56
Duration of AH (years)	10.77 ± 4.80
Duration of RAH (years)	4.12 ± 1.68
**Biochemical parameters**	
WBC (10^9^/L)	6.96 ± 1.94
HGB (g/dL)	13.44 ± 1.58
HCT (%)	40.35 ± 4.41
RDW (%)	13.91 ± 1.25
PLT (10^9^/L)	245.73 ± 64.33
Creatinine (mg/dL)	1.17 ± 0.85
eGFR (mL/min/1.73 m^2^)	70.57 ± 21.19
AST (U/L)	28.77 ± 8.32
ALT (U/L)	30.48 ± 9.81
INR	1.17 ± 0.31
APTT (seconds)	32.07 ± 3.97
UA (mg/dL)	6.86 ± 1.48
HbA1C (%)	6.86 ± 1.18
Fasting glucose (mg/dL)	121.65 ± 44.84
TSH (uIU/mL)	2.20 ± 2.26
TC (mg/dL)	197.78 ± 40.65
LDL (mg/dL)	126.49 ± 35.14
HDL (mg/dL)	46.89 ± 10.66
TG (mg/dL)	132.94 ± 65.60
**Comorbidities**	
Paroxysmal AF	21 (18.26%)
Persistent AF	3 (2.61%)
Permanent AF	4 (3.48%)
Hyperthyroidism	12 (10.43%)
Hypothyroidism	10 (8.70%)
COPD	5 (4.35%)
Smoking at admission	18 (15.65%)
Smoking history	25 (21.74%)
Diagnosed CKD	40 (34.78%)
CAD	80 (69.57%)
AMI history	17 (14.78%)
PCI history	39 (33.91%)
CABG history	9 (7.83%)
PAD	16 (13.91%)
Ischemic stroke history	13 (11.30%)
TIA history	21 (18.26%)
Hemorrhagic history	4 (3.48%)
HFpEF	94 (81.74%)
Hypercholesterolemia	80 (69.57%)
Albuminuria	12 (10.43%)
**Echocardiography parameters**	
LVEF (%)	59.72 ± 5.81
LA dimension in LAX (mm)	39.68 ± 3.77
LA surface area in Ap4CH (cm^2^)	21.19 ± 2.75
LA volume (mL)	94.73 ± 19.25
LAVI (mL/m^2^)	46.28 ± 10.89
LVs (mm)	44.50 ±4.24
LVd (mm)	49.38 ± 4.36
IVS (mm)	11.64 ± 0.97
PW (mm)	11.22 ± 1.02
LVMI (g/m^2^)	110.68 ± 21.68
RV (mm)	28.00 ± 2.34
LVMM (g)	216.22 ± 46.78
RWT	0.46 ± 0.05

Abbreviations: M—mean; SD—standard deviation; BMI—body mass index; AH—arterial hypertension, RAH—resistant arterial hypertension, WBC—white blood cells; HGB—hemoglobin; HCT—hematocrit; RDW—red cell distribution width; PLT—platelets; eGFR—estimated glomerular filtration rate; AST—aspartate aminotransferase; ALT—aminotransferase alanine; INR—international normalized ratio; APTT—activated partial thromboplastin time; UA—urine acid; HbA1C—glycated hemoglobin; TSH—thyroid stimulating hormone; TC—total cholesterol; LDL—low-density lipoprotein; HDL—high-density lipoprotein; TG—triglycerides; AF—atrial fibrillation; COPD—chronic obstructive pulmonary disease; CKD—chronic kidney disease; CAD—coronary artery disease; AMI history—acute myocardial infarction history; PCI history—percutaneous coronary intervention history; CABG history—coronary artery bypass graft history; PAD—peripheral arterial disease; TIA—transient ischemic attack; LVEF—left ventricle ejection fraction; LA—left atrium; LAX—long axis view; Ap4CH—apical four chamber; LAVI—left atrium volume index; LVs—left ventricle systolic; LVd—left ventricle diastolic; IVS—interventricular septum; PW—posterior wall; LVMI—left ventricle mass index, RV—right ventricle; LVMM—left ventricle muscle mass; RWT—relative wall thickness; HFpEF—heart failure with preserved ejection fraction.

**Table 2:** Pharmacotherapy of RAH.

Beta-Blockers	Percentage
Bisoprolol	33 (28.70%)
Nebivolol	47 (40.87%)
Betaxolol	4 (3.48%)
Metoprolol succinate	15 (13.04%)
Carvedilol	3 (2.61%)
Sotalol	8 (6.96%)
**Alpha-blockers**	
Doxazosin	45 (39.13%)
**Loop diuretics**	
Furosemide	9 (7.83%)
Torasemide	38 (33.04%)
**Thiazide diuretics**	
Hydrochlorothiazide	43 (37.39%)
Amiloryd + hydrochlorothiazide	1 (0.87%)
**Thiazide-like diuretics**	
Indapamide	33 (28.7%)
**Dihydropirydyne antagonist calcium**	
Lercanidipine	62 (53.91%)
Amlodipine	41 (35.65%)
Lacidipine	5 (4.35%)
Nitrendypine	4 (3.48%)
**Angiotensin-converting enzyme inhibitors**	
Ramipril	24 (20.87%)
Perindopril	18 (15.65%)
Lisinopril	5 (4.35%)
**AT1 receptor antagonists (sartans)**	
Telmisartan	37 (32.17%)
Candesartan	9 (7.83%)
Valsartan	17 (14.78%)
Olmesartan	2 (1.74%)
**Mineralocorticoid antagonist receptors**	
Spironolactone	93 (80.87%)
Eplerenone	10 (8.70%)
**Imidazoline receptor agonist**	
Clonidine	69 (60.00%)

**Table 3:** Baseline characteristics of patients with RAH without T2DM.

Variable	Patients with RAH without T2DM			
	Numbers	Mean	Median	Standard Deviation
Age (years)	62	65.1452	67.0000	13.19072
BMI (kg/m^2^)	62	30.1705	29.5450	4.70880
Duration of AH (years)	62	10.9194	10.0000	5.20812
Duration of RAH (years)	62	4.2903	4.0000	1.89380
HGB (g/dL)	62	13.6839	13.6500	1.48569
HCT (%)	62	40.7677	41.0500	4.28347
RDW (%)	62	13.7758	13.6000	1.02824
WBC (tys./uL)	62	7.1158	6.7750	2.06081
PLT (tys./mm^3^)	62	247.6290	244.5000	64.98177
Creatinine (mg/dL)	62	1.0503	1.0000	0.21059
eGFR (mL/min/1.73 m^2^)	62	73.1735	72.3500	18.10835
AST (U/L)	62	29.3548	29.0000	8.63105
ALT (U/L)	62	30.2903	31.5000	9.53060
INR	62	1.2258	1.1200	0.40694
APTT (seconds)	62	32.3694	31.6000	4.28633
UA (mg/dL)	62	6.6048	6.4650	1.26792
HbA1c (%)	62	6.1419	6.1200	0.40729
Fasting glucose (mg/dL)	62	100.9032	98.5000	12.64485
TSH (uIU/mL)	62	2.0874	1.9645	1.43933
TG (mg/dL)	62	191.6129	194.5000	38.26583
LDL (mg/dL)	62	123.2839	127.5000	33.61000
HDL (mg/dL)	62	46.9032	45.5000	9.94857
TG (mg/dL)	62	114.2742	102.0000	44.17223

**Table 4:** Baseline characteristic patients with RAH and T2DM.

Variable	Patients with RAH without T2DM			
	Numbers	Mean	Median	Standard Deviation
Age (years)	53	66.2830	66.0000	8.21672
BMI (kg/m^2^)	53	30.6787	29.7600	4.40255
Duration of AH (years)	53	10.5849	10.0000	4.32094
Duration of RAH (years)	53	3.9245	4.0000	1.38466
HGB (g/dL)	53	13.1642	13.1000	1.65461
HCT (%)	53	39.8585	39.7000	4.54463
RDW (%)	53	14.0849	13.6000	1.45766
WBC (tys./uL)	53	6.7747	6.8900	1.78032
PLT (tys./mm^3^)	53	245.6792	234.0000	64.17038
Creatinine (mg/dL)	53	1.3064	1.0600	1.22343
eGFR (mL/min/1.73 m^2^)	53	67.5260	65.5400	24.13409
AST (U/L)	53	28.0943	28.0000	7.95967
ALT (U/L)	53	30.6981	30.0000	10.21798
INR	53	1.1111	1.1000	0.10595
APTT (seconds)	53	31.7283	31.8000	3.56486
UA (mg/dL)	53	7.1506	6.9800	1.65397
HbA1C (%)	53	7.7068	7.5600	1.22287
Fasting glucose (mg/dL)	53	145.9283	136.0000	55.74022
TSH (uIU/mL)	53	2.3378	1.8760	2.96025
TC (mg/dL)	53	204.9981	211.0000	42.51014
LDL (mg/dL)	53	130.2377	143.0000	36.82362
HDL (mg/dL)	53	46.8736	45.0000	11.53877
TG (mg/dL)	53	154.7811	125.0000	79.01592

**Table 5:** Cardiovascular morbidity in the studied patient groups.

Parameters	Patients with RAH without T2DM (%)	Patients with RAH and T2DM (%)
Paroxysmal AF	14 (22.58%)	7 (13.20%)
Persistent AF	2 (3.22%)	1 (1.89%)
Chronic AF	3 (4.84%)	1 (1.89%)
HFrEF	0 (0.00%)	1 (1.89%)
HFmrEF	4 (6.45%)	1 (1.89%)
HFpEF	46 (74.19%)	48 (90.57%)
Previous stroke history	8 (12.90%)	9 (16.98%)
Previous ischemic stroke	6 (9.68%)	7 (13.20%)
Previous hemorrhagic stroke	2 (3.23%)	2 (3.78%)
Previous TIA	11 (17.74%)	10 (18.87%)
CAD	37 (59.68%)	43 (81.13%)
Previous PCI	15 (24.19%)	24 (45.28%)
Previous MI	5 (8.06%)	12 (22.64%)
Previous CABG	1 (1.61%)	8 (15.09%)

Abbreviations: AF—atrial fibrillation, HFrEF—heart failure with rejected ejection fraction, HFmrEF—heart failure with mild rejected ejection fraction, HFpEF—heart failure with preserved ejection fraction, TIA—transient ischemic attack, CAD—coronary artery disease, PCI—percutaneous coronary intervention, MI—myocardial infarction, CABG—coronary artery bypass graft.

**Table 6:** Echocardiographic parameters in patients with RAH without T2DM.

Variable	RAH without Diabetes			
	Numbers	Mean	Median	Standard Deviation
LVEF (%)	62	60.0323	61.5000	6.08672
LA dimension in LAX (mm)	62	39.4194	39.0000	4.11135
LA surface area in Ap4CH (cm^2^)	62	20.7484	20.5000	2.85380
LA volume (mL)	62	90.7358	89.0000	17.93869
LAVI (mL/m^2^)	62	44.7406	43.0000	9.82495
LVs (mm)	62	44.4516	44.5000	4.51837
LVd (mm)	62	49.1613	49.0000	4.52749
IVs (mm)	62	11.5323	12.0000	0.88183
PW (mm)	62	11.1613	11.0000	0.83359
LVMI (g/m^2^)	62	108.6935	106.5000	19.58135
RV (mm)	62	27.7903	28.0000	2.38257
LVMM (g)	62	211.8142	214.0000	45.32293
RWT	62	0.4565	0.4650	0.04774

**Table 7:** Echocardiographic parameters in patients with RAH and T2DM.

Variable	RAH with Diabetes			
	Numbers	Mean	Median	Standard Deviation
LVEF (%)	53	59.3585	60.0000	5.51255
LA dimension in LAX (mm)	53	40.0000	41.0000	3.33974
LA surface area in Ap4CH (cm^2^)	53	21.7245	21.7000	2.55583
LA volume (mL)	53	99.4057	99.0000	19.83242
LAVI (mL/m^2^)	53	48.0849	46.0000	11.85744
LVs (mm)	53	44.5472	45.0000	3.92999
LVd (mm)	53	49.6415	49.0000	4.18373
IVs (mm)	53	11.7736	12.0000	1.06774
PW (mm)	53	11.2830	11.0000	1.19900
LVMI (g/m^2^)	53	113.0000	106.0000	23.87789
RV (mm)	53	28.2453	28.0000	2.28629
LVMM (g)	53	221.3732	207.0000	48.34140
RWT	53	0.4560	0.4600	0.05267

Abbreviations for Table 6 and Table 7: LVEF—left ventricle ejection fraction, LA—left atrium, Ap4CH—apical four chamber, LVs—left ventricle systolic, LVd—left ventricle diastolic, IVs—interventricular septum systolic, PW—posterior wall, LVMI—left ventricle mass index, RV—right ventricle, LVMM—left ventricle muscle mass, RWT—relative wall thickness.

**Table 8:** Significance differences in expression values of the analyzed miRs (hsa-miR-1, hsa-miR-126*, hsa-miR-133a, hsa-miR-143, hsa-miR-145*, hsa-miR-155, hsa-miR-195, hsa-miR-208, hsa-miR-21, hsa-miR-26b, hsa-miR-320) in relation to the gender of the examined patients: results of statistical analysis.

Mann–Whitney U Test Relevant Variable: Gender Marked Results Are Significant with *p* < 0.05					
	Sum. of Ranks Women	Sum. of Ranks Men	U	Z	*p*
LogRQ hsa-miR-1	3000.000	2356.000	1275.000	0.238814	0.811250
LogRQ hsa-miR-126*	3100.000	2465.000	1330.000	−0.174380	0.861567
LogRQ hsa-miR-133a	3261.000	2410.000	1282.000	0.664569	0.506327
LogRQ hsa-miR-143	3232.000	2439.000	1311.000	0.480143	0.631126
LogRQ hsa-miR-145*	3124.000	2336.000	1255.000	0.517047	0.605124
LogRQ hsa-miR-155	3106.000	2565.000	1336.000	−0.321155	0.748093
LogRQ hsa-miR-195	3147.000	2524.000	1377.000	−0.060415	0.951825
LogRQ hsa-miR-208	3001.000	2459.000	1290.000	−0.287976	0.773366
LogRQ hsa-miR-21	3150.000	2521.000	1380.000	−0.041337	0.967027
LogRQ hsa-miR-26b	3155.000	2516.000	1385.000	−0.009539	0.992389
LogRQ hsa-miR-320	3164.000	2507.000	1379.000	0.047696	0.961958

## Data Availability

Data are contained within the article.
